# Fear of Cancer Recurrence Contributes Largely to Patient Anxiety and Depression and Quality of Life in a Prospective Cohort of Chinese Breast Cancer Patients for Postoperative Radiotherapy

**DOI:** 10.1155/tbj/5788053

**Published:** 2025-12-22

**Authors:** Zhensheng Li, Yunjiang Liu, Yue Li, Yuguang Shang, Jun Zhang, Xiaohui Ji, Zhiping Zhao, Xuejuan Duan, Wenhui Geng, Junpu Yin

**Affiliations:** ^1^ Department of Radiation Oncology, The Fourth Hospital of Hebei Medical University, Shijiazhuang, China, hebmu.edu.cn; ^2^ Department of Breast Surgery, The Fourth Hospital of Hebei Medical University, Shijiazhuang, China, hebmu.edu.cn; ^3^ Department of Ultrasound, The Fourth Hospital of Hebei Medical University, Shijiazhuang, China, hebmu.edu.cn

**Keywords:** anxiety and depression, breast cancer, Chinese women, fear of cancer recurrence, quality of life

## Abstract

**Background:**

Fear of cancer recurrence (FCR) is common among Chinese breast cancer (BC) patients following surgery, chemotherapy, and radiotherapy (RT). Understanding the prevalence and impact of FCR, particularly during the RT period, on anxiety, depression, and quality of life may inform strategies to manage patient distress.

**Methods:**

From July 2015 to December 2016, 486 women undergoing RT for BC at the Fourth Hospital of Hebei Medical University, China, were prospectively enrolled in the study. Anxiety, depression, and quality‐of‐life changes were assessed using the Hospital Anxiety and Depression Scale (HADS) and the European Organization for Research and Treatment of Cancer’s Quality‐of‐Life questionnaires (QLQ‐C30, QLQ‐BR23) before and after RT. FCR was assessed using a modified question from the QLQ‐BR23, *“how much have you worried about tumor recurrence (over the last week),”* which demonstrated the satisfactory construct validity and reliability in a prior pilot study. ANOVA, multivariate linear regressions, and ordinal logistic regressions were performed to evaluate odds ratio (OR) and *p* values. Pain and sleep disturbance were included as covariates in secondary models.

**Results:**

Of the 486 women enrolled, 23 (4.7%) patients declined participation. Of the 463 analyzed patients, the mean age was 47 years old. 386 patients (83.4%) elected for mastectomies. FCR levels prior to RT were reported as “*none*” (29.4%), “*a little bit*” (51.2%), “*some*” (12.1%), and “*very much*” (7.3%). Increased FCR severity was associated with elevated median anxiety score (1.5, 5.0, 7.0, 8.5) and increased rates of clinically significant anxiety (anxiety score ≥ 11; 0%, 3.4%, 12.5%, 26.5%). Similarly, median depression scores (2.0, 4.0, 6.0, 6.5) rose with FCR severity, accompanied by higher prevalence of atypical depression (depression score ≥ 11; 2.2%, 3.4%, 5.4%, 17.7%) (all *p* < 0.001). Compared to the reference “*none,”* each unit increase of FCR severity was independently associated with one category (“*normal*,” “*borderline*,” and “*abnormal*”) of increased anxiety with OR (*p*) of 2.593 (*p* = 0.011), 5.889 (*< 0.001*), and 14.621 (*< 0.001*) and increased depression with OR (*p*) of 2.406 (*0.008*), 3.045 (*0.009*), and 7.210 (*< 0.001*), respectively. FCR severity was negatively associated with most quality‐of‐life domains (*p* < 0.05). Similar associations persisted post RT.

**Conclusions:**

FCR is a significant contributor to psychological distress and reduced quality of life among Chinese breast cancer patients during the RT period. Routine screening for anxiety and depression and targeted intervention for FCR should be prioritized in this population.

## 1. Introduction

Fear of cancer recurrence (FCR) is defined as “*fear, worry, or concern relating the possibility that cancer will come back or progress*” [1, 2]. While a certain level of FCR is considered a normal psychological response, excessive or persistent FCR can significantly elevate psychological stress, impair physical and emotional functioning, and negatively impact quality of life [2–6].

For reasons not fully understood, FCR tends to be more prevalent, persistent, and severe among breast cancer patients compared to other cancer populations [7, 8]. Among races, Asian, and particularly Chinese, women with breast cancer have been reported to experience higher incidence and prevalence of FCR than their counterparts of other racial or ethnic backgrounds [8–10].

In China, women with breast cancer are often diagnosed at a younger age, present with larger tumors and more lymph node involvement, and are more likely to undergo mastectomy rather than breast‐conserving surgery, compared to patients in the United States and Europe [8, 9]. These factors may contribute to the heightened frequency and severity of FCR in Chinese women with breast cancer.

FCR is a known driver of psychological distress, frequently manifesting as anxiety and depression [3]. Emotional distress is commonly exacerbated by surgery, chemotherapy, radiotherapy (RT), and their associated side effects [1, 6]. Given the growing evidence supporting the efficacy of psychosocial interventions to manage FCR, it is critical to understand how FCR emerges and influences patient anxiety, depression, and quality of life, particularly during the period of postoperative RT, which can be a physically and emotionally taxing time for patients. We propose that this RT period also represents an important window for identification of patients at risk and intervention if necessary. Routine screening for FCR during RT may allow timely referral to mental health professionals, potentially improving long‐term psychological outcomes and reducing patients’ overall healthcare burden.

With these considerations in mind, we designed a prospective study to examine the prevalence and impact of FCR among Chinese breast cancer women during postoperative RT. We believe these studies not only deepen our understanding of FCR in this population but also support the development of more personalized approaches to screening, prevention, and intervention against FCR for Chinese breast cancer patients globally [10–12].

## 2. Methods

### 2.1. Study Design and Data Collection

Between July 2015 and December 2016, all women with unilateral breast cancer admitted for postoperative RT at the Fourth Hospital of Hebei Medical University in China were screened for study enrollment. Eligible patients met the following inclusion criteria: (1) age 18 years or older at the time of breast cancer diagnosis; (2) within 180 days of completing surgery and four to six cycles of adjuvant chemotherapy; (3) scheduled to receive RT to the chest wall/breast and supraclavicular/subclavian lymph node regions; and (4) able to complete study questionnaires with minimal instructions. Patients were excluded if they had (1) history of other malignant tumors; (2) comorbidities such as poorly controlled diabetes, cardiovascular disease, or disorders of the liver, kidneys, or immune system; (3) history of psychological or mental illness; or (4) experienced a major life event within the past 6 months such as divorce, serious physical injury, or the death of a close family member or friend.

The Chinese version of the Hospital Anxiety and Depression Scale (HADS) and the European Organization for Research and Treatment of Cancer (EORTC) QLQ‐C30 (C30) and QLQ‐BR23 (BR23) questionnaires were selected to measure anxiety, depression, and quality of life within ±1 day of the initiation and completion of RT. These questionaries in Chinese have previously been validated and are commonly used in clinics and research in China. To better assess FCR specifically, additional explanatory text was added in parentheses to one question in the BR23 questionnaire to improve patient comprehension. The modified question’s construct validity and reliability were tested with satisfactory statistical performance in a pilot study prior to the main study.

The pilot study consisted of 56 breast cancer patients who met the same eligibility criteria as the main study recruited in the same department. Participants completed a questionnaire containing the modified single‐item FCR question alongside the validated Chinese version of the Fear of Cancer Recurrence Inventory‐Short Form (FCRI‐SF). Questionnaires were administered twice over a 4‐week interval between January 2015 and March 2015. The modified question about FCR demonstrated strong concurrent validity with the two FCRI‐SF total scores over 4 weeks (Spearman’s *r* = 0.830 and 0.865, *p* = 0.013 and *0.009,* respectively). Significant correlations were also found between the modified single question and FCRI‐SF items 1–9 at both timepoints (all *p*  < 0.050). Additionally, the modified question showed high test–retest reliability over the 4‐week period (Spearman’s *r* = 0.783, *p* = 0.031).

This study was approved by the Research Ethics Committee of the Fourth Hospital of Hebei Medical University (#2015‐166). All participants provided written informed consent prior to study enrollment.

### 2.2. Questionnaires and Variables

The C30 consists of 30 item questions (C30‐Q1–C30Q30). Questions C30‐Q6–C30‐Q28 assess the frequency of specific symptoms or complaints experienced over the past week, using a four‐point scale with response choices of “*none*,” “*a little bit*,” “*some*,” or “*very much*.” Specifically, C30‐Q9 (“*have you ever had pain?*”), C30‐Q11 (“*have you ever had difficulty sleeping?*”), and C30‐Q22 (“*have you ever felt worried about anything*”) were about pain and sleep disturbance.

The BR23 consists of 23 item questions (BR30‐Q1–BR30‐Q23) specific to breast cancer. Most questions refer to experiences in the past week except for BR23‐Q14 to BR23‐Q16, which address aspects of sexual life in the past 4 weeks. To assess FCR severity, we used a slightly modified version of BR23‐Q13 in Chinese: “*how much have you worried about illness (mainly about tumor recurrence)?”*. This item retained the original response format used across the BR23: “*none*,” “*a little bit*,” “*some*,” or “*very much*.”

The HADS consists of seven item questions assessing anxiety and depression “*in the past week*.” All responses are scored on a four‐point Likert scale (0–3). Total scores were calculated using standard score formulas and category definitions provided by HADS’ developer. Based on total scores, anxiety and depression levels were categorized as “*normal*” (score 0–7), “*borderline*” (8–10), and “*abnormal*” (11–21). The Chinese version of HADS has been validated among Chinese breast cancer patients with good reliability estimates of Cronbach’s α over 0.80 for both subscales [13]. The choice of cutoff scores for categorization was supported in the literature [14]. In this study, the HADS demonstrated excellent internal consistencies for anxiety (Cronbach α = *0.87*) and depression (Cronbach α = *0.80*) subscales.

Quality‐of‐life functioning domain scores were calculated according to the official scoring manuals provided by the EORTC, using item responses from the C30 and BR23 questionnaires.

### 2.3. Statistical Methods

Continuous and categorical variables were summarized with descriptive statistics. No missing values were imputed. Chi‐squared and Kruskal–Wallis one‐way ANOVA tests were used for comparison or relationship evaluation. Effects of binary and ordered FCR severity as main explanatory variables were estimated through multivariate binary and ordinal logistic regression models. Odds ratio (OR), along with corresponding 95% confidence interval (CI) and *p* value, was reported. Covariates in the final multivariate models were selected based on univariate analysis results and clinical prospectives. A two‐sided *p* value *< 0.05* was considered statistically significant. All statistical analyses were performed using SAS 9.4 for Windows.

## 3. Results

### 3.1. Baseline Characteristics

A total of 486 women were enrolled in the study. Of these, 23 (4.7%) patients later declined participation. The remaining 463 women who completed the study questionnaires were included in the analysis. Table 1 summarized their baseline characteristics, defined at the time of study enrollment. At baseline, 51.2%, 12.1%, and 7.3% of patients reported experiencing FCR “*a little bit*,” “*some,”* and “*very much*,” respectively. Following RT, these statistics were 55.1%, 12.2%, and 9.5%, respectively.

**Table 1:** Characteristics of patients at baseline.

Variable	Class	*n*	Mean ± std	Percent	Median (Q1–Q3)	Minimum–Maximum
Age, years		463	47.2 ± 9.56	*100*	48.0 (42.0–54.0)	19–89
BMI, kg/m^2^		463	25.9 ± 3.33	*100*	25.9 (23.7–28.0)	14.8–39.1
Breast with tumor	Left	234		*50.5*		
	Right	229		*49.5*		
TNM stage	I	51		*11.0*		
	II	177		*38.2*		
	III	221		*47.7*		
	IV	14		*3.0*		
Surgery	BCS	77		*16.6*		
	Mastectomy	386		*83.4*		
ALND	No	62		*13.4*		
	Yes	401		*86.6*		
Education	Illiteracy	15		*3.2*		
	Elementary	73		*15.8*		
	Junior high school	203		*43.8*		
	High school	101		*21.8*		
	College and above	71		*15.3*		
FCR severity	“*none”*	136		*29.4*		
	“*a little bit”*	237		*51.2*		
	“*some”*	56		*12.1*		
	“*very much”*	34		*7.3*		
HAD score	Anxiety	451	4.9 ± 3.35		5.0 (2.0–7.0)	0.0–20.0
	Depression	454	4.5 ± 3.23		4.0 (2.0–6.0)	0.0–18.0
Anxiety level	“*normal”*	373		*80.6*		
	“*borderline”*	54		*11.7*		
	“*abnormal”*	24		*5.2*		
	*missing*	12		*2.6*		
Depression level	“*normal”*	373		*80.6*		
	“*borderline”*	61		*13.2*		
	“*abnormal”*	20		*4.3*		
	*missing*	9		*1.9*		
Pain	“*none”*	173		*37.4*		
	“*a little bit”*	239		*51.6*		
	“*some”*	49		*10.6*		
	“*very much”*	2		*0.0*		
Sleep disturbance	“*none”*	192		*41.5*		
	“*a little bit”*	182		*39.3*		
	“*some”*	65		*14.0*		
	“*very much”*	24		*5.2*		

*Note:* The italic values under “Percent” in the table referred to the percents of patients who had the feature of subcategory under the category labeled. Std, standard deviation; Q1, Crst quartile; Q3, third quartile.

Abbreviations: ALND, axillary lymph node dissection; BCS, breast‐conserving surgery; BMI, body mass index; FCR, fear of cancer recurrence; HADS, Hospital Anxiety and Depression Scale.

### 3.2. Relationships of FCR, Pain, and Sleep Disturbance With Anxiety and Depression Before RT

Table 2 summarizes the results. FCR severity was significantly associated with both anxiety and depression scores, similar to the associations, which both pain and sleep disturbance were observed to have (all *p* < 0.001). Figure 1 illustrates the near‐linear relationships between FCR severity and both anxiety and depression scores. When analyzed categorically, these relationships also remained significant (all *p* < 0.001*;* Table 3). Similar relationships were observed in the post‐RT data.

**Table 2:** Relationships of anxiety and depression scores with FCR, pain, and sleep disturbance before RT (*N* = 463).

Variable	Class	HADS (?) score on anxiety					HADS (?) score on depression				
		*n*	Mean ± std	Median	*F* ^a^	*p* ^a^	*n*	Mean ± std	Median	*F* ^a^	*p* ^a^
FCR	“*none”*	130	2.26 ± 2.47	1.5	146.8	*< 0.001*	135	3.03 ± 2.88	2.0	70.1	*< 0.001*
	“*a little bit”*	231	5.34 ± 2.59	5.0			229	4.54 ± 2.92	4.0	229	
	“*some”*	56	7.16 ± 3.17	7.0			56	5.96 ± 3.09	6.0	56	
	“*very much”*	34	8.44 ± 3.96	8.5			34	7.5 ± 3.63	6.5	34	
Pain	“*none”*	166	3.69 ± 2.99	3.5	43.5	*< 0.001*	170	3.53 ± 3.02	3.0	35.9	*< 0.001*
	“*a little bit”*	234	5.35 ± 3.17	6.0			233	4.80 ± 3.15	5.0		
	“*some and very much”*	51	6.86 ± 3.85	6.0			51	6.25 ± 3.34	6.0		
Sleep	“*none”*	186	3.98 ± 2.92	4.0	35.3	*< 0.001*	189	3.72 ± 2.83	3.0	29.9	*< 0.001*
Disturbance	“*a little bit”*	177	4.95 ± 2.93	5.0			179	4.50 ± 3.11	4.0		
	“*some”*	64	6.33 ± 3.71	6.5			62	5.56 ± 2.93	5.5		
	“*very much”*	24	8.00 ± 5.03	7.5			24	7.67 ± 4.89	6.5		

*Note:* *p* value (in italics) are from the Kruskal–Wallis ANOVA test.

Abbreviations: FCR, fear of cancer recurrence; HADS, Hospital Anxiety and Depression Scale; RT, radiotherapy; std, standard deviation.

^a^From the Kruskal–Walis ANOVA analysis as both anxiety and depression scores were not normally distributed.

Figure 1Relationships of severity of FCR with anxiety and depression scores before RT.

**Figure:**
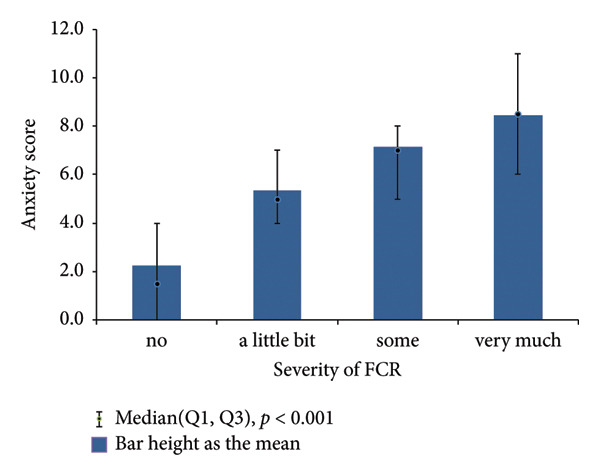
(a)

**Figure:**
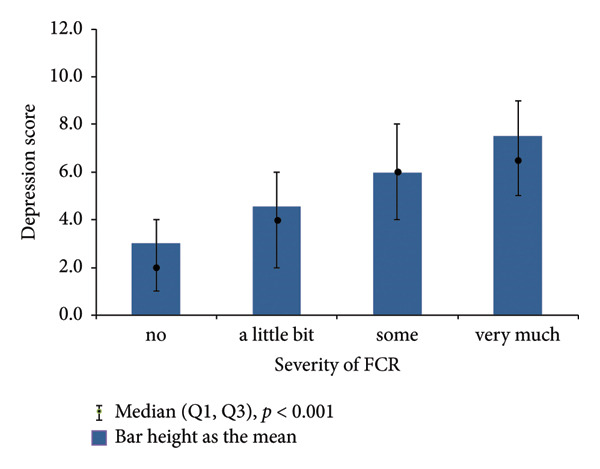
(b)

**Table 3:** Relationship of anxiety and depression levels with FCR, pain, and sleep disturbance before RT.

Variable	Class	Anxiety level (*n*, %)				*p* ^a^	Depression level (*n*, %)				*p* ^a^
		Normal	Borderline	Abnormal	Missing		Normal	Borderline	Abnormal	Missing	
FCR	“*none”*	126 (92.7)	4 (2.9)		6 (4.4)	*< 0.001*	122 (89.7)	10 (7.4)	3 (2.2)	1 (0.7)	*< 0.001*
	“*a little bit”*	197 (83.1)	26 (11.0)	8 (3.4)	6 (2.5)		191 (80.6)	30 (12.7)	8 (3.4)	8 (3.4)	
	“*some”*	36 (64.3)	13 (23.2)	7 (12.5)			41 (73.2)	12 (21.4)	3 (5.4)		
	“*very much”*	14 (41.2)	11 (32.4)	9 (26.5)			19 (55.9)	9 (26.5)	6 (17.7)		
Pain	“*none”*	152 (87.9)	11 (6.4)	3 (1.7)	7 (4.1)	*< 0.001*	151 (87.3)	13 (7.5)	6 (3.5)	3 (1.7)	*0.014*
	“*a little bit”*	189 (79.1)	33 (13.8)	12 (5.0)	5 (2.1)		188 (78.7)	35 (14.6)	10 (4.2)	6 (2.5)	
	“*some* and *very much”*	32 (62.8)	10 (19.6)	9 (17.7)			34 (66.7)	13 (25.5)	4 (7.8)		
Sleep	“*none”*	169 (88.0)	12 (6.3)	5 (2.6)	6 (3.1)	*< 0.001*	168 (87.5)	16 (8.3)	5 (2.6)	3 (1.6)	*< 0.001*
Disturbance	“*a little bit”*	148 (81.3)	26 (14.3)	3 (1.7)	5 (2.8)		144 (79.1)	29 (15.9)	6 (3.3)	3 (1.7)	
	“*some*”	44 (67.7)	11 (16.9)	9 (13.9)	1 (1.5)		47 (72.3)	13 (20.0)	2 (3.1)	3 (4.6)	
	“*very much”*	12 (50.0)	5 (20.8)	7 (29.2)			14 (58.3)	3 (12.5)	7 (29.2)		

*Note:* These *p* values are from the Chi‐square tests. If any *p* value is less than 0.05, it indicates that there is statistically significant relationship between the categorical variable (FCR, Pain, Sleep Disturbance) and another categorical variable (Anxiety level, Depression level) analyzed among these patients who have the non‐missing values of both variables.

Abbreviations: FCR, fear of cancer recurrence; RT, radiotherapy.

^a^ *p* from the Chi‐square test in patients with nonmissing value.

### 3.3. Binary and Ordinal Logistic Regression of Anxiety and Depression on FCR

Anxiety and depression scores were not normally distributed. Therefore, logistic regression models were applied to score‐based categorical outcomes. Two outcome types were analyzed: a binary variable (normal/abnormal) and an ordinal variable (normal/borderline/abnormal). Prior to ordinal logistic regression modeling, both ordinal variables as outcomes were confirmed to hold the proportional odd assumption.

Table 4 presents the regression results. Compared to patients reporting with “*none”* or “*a little bit”* of FCR, patients with “*some”* and “*very much”* FCR had 5.727 (*p* = 0.002) and 24.114 (*p*  < 0.001) times higher risk of having “*abnormal”* anxiety and 1.658 (*p* = 0.470) and 10.122 (*p*  < 0.001) times higher risk of having “*abnormal”* depression, respectively. Multivariate ordinal logistic regression models showed that compared to patients with “*none*” FCR, patients with one level increase of FCR severity were significantly associated with having a one‐category increase (“*normal*” to “*borderline*” to “*abnormal*”) in anxiety with estimated OR (*p*) as 2.593 (*0.011*), 5.889 (*< 0.001*), 14.621 (*< 0.001*), and in depression with estimated OR (*p*) as 2.406 (*0.008*), 3.045 (*0.009*), and 7.210 (*< 0.001*). These relationships were clearly demonstrated here to be independent and significant.

**Table 4:** Logistic regression model before RT.

Regression model		Class	Univariate			Multivariate^a^		
Endpoint	Variable		OR	95% CI	*p*	OR	95% CI	*p*
Binary^b^								
Anxiety	FCR	“*none*/*a little bit”*	1.000		*ref.*	1.000		*ref.*
		“*some”*	6.303	(2.189–18.147)	*0.001*	5.727	(1.923–17.054)	*0.002*
		“*very much”*	15.884	(5.642–44.722)	*< 0.001*	24.114	(7.532–77.209)	*< 0.001*
Depression	FCR	“*none*/*a little bit”*	1.000		*ref.*	1.000		*ref.*
		“*some”*	1.816	(0.491–6.724)	*0.371*	1.658	(0.421–6.524)	*0.470*
		“*very much”*	6.877	(2.367–19.979)	*< 0.001*	10.122	(2.961–34.602)	*< 0.001*
Ordinal^c^								
Anxiety	FCR	“*none”*	1.000		*ref.*	1.000		*ref.*
		“*a little bit”*	2.482	(1.209–5.095)	*0.013*	2.593	(1.247–5.395)	*0.011*
		“*some”*	6.406	(2.783–14.747)	*< 0.001*	5.889	(2.506–13.839)	*< 0.0001*
		“*very much”*	14.693	(6.004–35.956)	*< 0.001*	14.621	(5.785–36.956)	*< 0.0001*
Depression	FCR	“*none”*	1.000		*ref.*	1.000		*ref.*
		“*a little bit”*	2.131	(1.126–4.033)	*0.020*	2.406	(1.252–4.622)	*0.008*
		“*some”*	3.028	(1.346–6.812)	*0.007*	3.045	(1.321–7.019)	*0.009*
		“*very much”*	6.677	(2.834–15.732)	*< 0.001*	7.210	(2.950–17.623)	*< 0.001*

*Note:* *p* value from the logistic regression analysis. RT, radiotherapy; ref., reference.

Abbreviations: CI, confidence interval; FCR, fear of cancer recurrence; OR, odd ratio.

^a^Covariates included age, BMI, education, breast cancer laterality, TNM stage, surgery type of breast, and ALND.

^b^The “*abnormal”* one.

^c^The “*normal*,” “*borderline*,” and “*abnormal”* ones.

Considering the close relationships observed of pain and sleep disturbance with anxiety and depression (Tables 2 and 3), we conducted the same logistic regression models with them as additional covariates. Multivariate models showed the similar OR (*p*) of FCR as above. The consistent results indicated that the significant relationships of FCR with anxiety and depression could not be explained away by pain and sleep disturbance.

The same analyses were conducted using post‐RT data. Multivariate models without adjustment for pain and sleep disturbance showed that compared to patients with “*none*” of FCR, a patient with one level increase of FCR severity was significantly associated with a one‐category increase (“normal” to “borderline” to “abnormal”) of anxiety with estimated OR (*p*) as 16.552 (*0.001*), 49.466 (*< 0.001*), and 116.048 (*< 0.001*) and of depression with estimated OR (*p*) as 3.731 (*0.001*), 15.866 (*< 0.001*), and 28.637 (*< 0.001*). It appeared that these relationships were stronger than those generated from using data prior to RT. Adding pain and sleep disturbance in multivariate models did not substantially alter these findings, further confirming independent and significant relationships of FCR with anxiety and depression at the end of RT.

Finally, although breast cancer stage III/IV (vs. stage I) and patient age < 35 (vs. age ≥ 65) years demonstrated to be associated with higher levels of anxiety and depression (ORs 1.213–3.205, *p* = 0.026 − 0.043) in univariate analysis before RT, they were not significant factors in all multivariate analyses or any analysis at the end of RT. The covariates of education, BMI, breast cancer laterality, breast surgery type, and ALND were not found to be significant factors associated with anxiety or depression level in either univariate or multivariate analyses before RT and at the end of RT.

### 3.4. Changes of FCR and Anxiety and Depression Over RT

A significant relationship was observed between two FCR severity measurements before and after RT (*X* ^2^ = 189.5, *p* = 0.001). Although the proportions of FCR severity changed little, both anxiety and depression scores had a slight increase only. The anxiety score [mean ± std (median)] increased from 4.91 ± 3.35 (5.0) to 5.35 ± 3.33 (5.0), while the depression score rose from 4.49 ± 3.23 (4.0) to 5.21 ± 3.76 (5.0). The paired differences in scores [mean ± std (median)] were 0.46 ± 2.83 (0.0) for anxiety and 0.67 ± 3.20 (0.0) for depression. As both paired score differences were not normally distributed, Wilcoxon sighed rank tests were conducted, revealing statistically significant increases in the median scores for both anxiety (*p* = 0.0005) and depression (*p*  <  0.0001). Assessing the relationship between changes in FCR and corresponding changes in anxiety and depression were beyond the scope of this study.

### 3.5. FCR Negatively Associates With Most Quality‐of‐Life Measures

The majority of quality‐of‐life functioning scores before and after RT were not normally distributed. Therefore, Kruskal–Wallis ANOVA tests were used to assess how quality‐of‐life scores were related to the FCR severity. Before RT, these H statistics (*p* value) for the specified quality‐of‐life functioning domains were as follows: physical functioning 29.5 (*p*  < 0.001), role functioning 46.9 (*p*  < 0.001), emotional functioning 133.9 (*p*  <  0.001), cognitive functioning 70.4 (*p*  < 0.001), social functioning 88.1 (*p*  < 0.001), global health functioning 66.8 (*p*  <  0.001), and body image functioning 185.6 (*p*  < 0.001). In contrast, associations with sexual functioning 4.6 (*p* = 0.205) and sexual enjoyment functioning 0.8 (*p* = 0.858) were not statistically significant. Similar patterns were observed after RT. When these findings were interpreted alongside the median of quality‐of‐life functioning domain scores, consistent inverse relationships between FCR severity and most quality‐of‐life scores were evident both before and after RT. Given the multifactorial and interdependent nature of quality‐of‐life domains, multivariate analyses were not pursued further.

## 4. Discussion

FCR is closely associated with anxiety, depression, and reduced quality of life in patients with cancer [1–3]. However, its specific manifestation and relationship to these psychosocial outcomes among Chinese women with breast cancer, particularly during RT, have not been well characterized. These patients have typically just completed surgery and cycles of chemotherapy, which are both major distress‐inducing events. In this large prospective study, we found that 7.3%–9.5% of these patients reported experiencing e FCR “*very much”* “*in the last week*.” Moreover, FCR severity showed significant relationships with anxiety, depression, and most quality‐of‐life functioning domains.

Although our study results cannot be generalized into cause–effect relationships, our findings clearly support FCR as an independent contributor to psychological distress and diminished quality of life. As RT typically represents the final phase of primary breast cancer treatment and spans 3–5 weeks prior to transitioning to the surveillance phase, we propose that the RT period is a strategically valuable time for screening and intervention against FCR in Chinese breast cancer patients.

FCR is a biopsychosocial event influenced by numerous factors [3, 12]. Many studies have showed its incidence and severity to be associated with race, age, education, cancer type, and side effects from procedures or treatments [11, 12, 15–17]. FCR is generally assessed using standardized patient‐reported questionnaires, which are usually not tailored to specific types of cancer or ethnicities. To date, a few clinical studies have focused on FCR in Chinese breast cancer patients [10, 17]. Ng et al. [17] investigated FCR in a subgroup of 173 Chinese breast cancer patients in Hong Kong using the Chinese version of the FCRI‐SF questionnaire. The FCRI‐SF consisted of nine‐item questions evaluating FCR over the past month on a five‐point Likert frequency scale (0 = “*not at all*”; 4 = “*a great deal or all the time*”) [18, 19]. In Ng’s study, FCRI‐SF showed a high internal consistency (Cronbach α = *0.87*) [17]. During the 8‐week postsurgery of these patients, their FCR prevalence rates at the levels of “*nonclinical”* (FCRI‐SF score < 13), “*subclinical”* (13–21), and “*clinical*” (> 21) were 54.3%, 31.2%, and 14.5%, respectively [17].

Direct comparisons between FCR studies are limited by differences in study populations, instruments, and timing; however, most reports have demonstrated that Chinese women with breast cancer exhibited a very higher prevalence of FCR compared to other racial groups [18, 19]. In our study, FCR was assessed using a single‐item question about FCR concerns “*in the last week*,” asked before and after RT. A pilot study supported the validity of this single‐item format of FCR assessment. This approach was chosen over the FCRI‐SF, which reflects the patient’s experience of FCR “*in the past month*,” to capture short‐term fluctuations, which are glossed over with a month‐to‐month measurement. And finally, given the constraints in a public hospital setting such as in China, where resources are limited and staff are overburdened, a brief single‐item questionnaire of FCR may be more practical for routine clinical use.

While identifying the predictors of FCR severity was beyond the scope of this analysis, our findings showed that FCR severity was significantly related to anxiety and depression, which were consistent with other studies [6, 20, 21]. However, the near‐linear relationships observed in our cohort were unexpected and have a great potential value in clinical practice. First, our single‐item FCR question, given its direct relationship linearly predicting depression and anxiety, could serve a good surrogate marker for broader psychological distress, potentially replacing distress thermometer (DT), which has shown mixed performance among non‐Western cancer patient settings [22, 23]. Second, a short FCR screening process could quickly identify some Chinese breast cancer women who are in need of psychological support, enabling timely specialist interventions [24–27]. Third, the RT duration could be the best time to manage any FCR‐related psychological and psychiatric issues for breast cancer patients. These patients have just finished surgery and chemotherapy and are facing or experiencing RT with fear. All of them are known causes of distress. Finally, oncologists should proactively collaborate with primary care providers in managing cancer‐related distress, especially in Chinese breast cancer women where psychiatric resources are limited.

Like other studies, our study found FCR to be associated with a lower quality of life in breast cancer patients [3, 20, 28]. Simard et al. [3] conducted a systematical review of FCR on quality of life in adult cancer survivors from 11 studies and found that low or less FCR had a good predictive power for high emotional or mental functioning but not for any level of physical and social performance. Ashing et al. [28] prospectively followed 137 Asian‐American breast cancer survivors and found that a higher baseline FCR predicted a lower physical, emotional, and functional well‐beings and breast cancer‐related quality of life (all *p*  < 0.01) after 1 year. In their study, FCR was assessed with a single question—“*I worry about my cancer coming back or spreading*”—with five response choices of 0 (*not at all*) to 4 (*very much*). This item was incorporated in the Functional Assessment of Cancer Therapy‐Breast (FACT‐B) questionnaire in the study. In our review, this single item was very similar to the modified BR23‐Q13 item used in our study. While we did not perform multivariate analyses to investigate the independent effect of FCR on quality‐of‐life functioning domains, the correlation between FCR and reduced quality of life in breast cancer patients was nonetheless evident and clinically meaningful for our population of Chinese women experiencing breast cancer treatment.

### 4.1. Study Strengths and Limitations

The strengths of this study include several key aspects. First, it was a large prospective study specifically examining FCR in Chinese breast cancer patients including during the RT period. Second, our analysis accounted for many potential confounding variables in the relationship between FCR, anxiety, and depression through multivariate models; additional models including pain and sleep disturbance further confirmed the independent contribution of FCR. Third, both continuous and categorical measures of anxiety and depression were analyzed, increasing the robustness of results. Fourth, the single‐item FCR question used in this study had been validated before its incorporation into the BR23 instrument, supporting its reliability and practical utility in clinical setting. However, we acknowledge the limitations of our study. Notably, FCR, anxiety, and depression were not assessed by clinicians and discrepancies in recall timeframes—for example, comparing experiences “*in the past 4 weeks”* versus “*in the past week*” may have introduced memory bias. In addition, the cross‐sectional nature of the data limits the ability to infer causality between FCR and psychological outcomes. There could be selection biases that potentially existed in a single‐center retrospective study. And finally, the generalizability of our findings to populations outside China remains uncertain and warrants further investigation. Nonetheless, in our view, these limitations do not substantially alter the overall conclusions of our study.

### 4.2. Clinical Implications

The high prevalence of FCR during the RT period in Chinese women with breast cancer women warrants clinical attention. The observed significant relationships of FCR, anxiety, depression, and reduced quality of life underscore the importance of timely assessment and intervention of FCR in this race population. A single‐item FCR question may serve as a practical and reliable surrogate for broader psychological distress in this population, offering a feasible tool for use in busy clinical settings.

## 5. Conclusion

FCR significantly contributes to psychological distress and is closely associated with lower quality of life among Chinese women with breast cancer undergoing RT. Routine screening and timely intervention for FCR in this population should be strongly recommended during the RT period to improve overall mental well‐being and support comprehensive cancer care.

**Nomenclature**
ALND: Axillary lymph node dissection
BC: Breast cancer
BCS: Breast‐conserving surgery
CI: Confidence interval
DT: Distress Thermometer
EORTC: European Organization for Research and Treatment of Cancer
FACT‐B: Functional assessment of cancer treatment for breast cancer
FCR: Fear of cancer recurrence
FCRI‐SF: Fear of cancer recurrence inventory‐short form
HADS: Hospital anxiety and depression scale
OR: Odds ratio
RTs: Radiotherapy

## Ethics Statement

This study was approved by the Research Ethics Committee of the Fourth Hospital of Hebei Medical University (FHHBU 2015‐166) in China. All patients were provided with the written informed consent before study enrollment.

## Disclosure

A preprint of its earlier version has previously been published online in Research Square [29]. The author list has slightly changed based on current contribution for the revised manuscript. All authors agreed with this change.

## Conflicts of Interest

The authors declare no conflicts of interest.

## Author Contributions

Z.L. supervised this study. Z.L., Y.L., and Y.L. were principally responsible for study design, analysis, and manuscript writing. Y.S., J.Z., X.J., Z.Z., X.D., W.G., and J.Y. were responsible for data collection, quality control, and partially contributed to the analysis. Y.L. contributed significantly to discussion and manuscript revision.

## Funding

The authors declared that partial financial supports were received from 2024 Hebei Provincial Health Commission’s Health Innovation Special Project (Item No: 242W7701Z) on the study for its data management, analysis, and manuscript writing and revision.

## Data Availability

The deidentified analysis datasets can be available from the corresponding author once the manuscript has been accepted for publication with the approval of the Fourth Hospital of Hebei Medical University in China.
